# Northern range shift may be due to increased competition induced by protection of species rather than to climate change alone

**DOI:** 10.1002/ece3.4348

**Published:** 2018-07-24

**Authors:** Loïc Marion, Benjamin Bergerot

**Affiliations:** ^1^ UMR CNRS ECOBIO Université Rennes Rennes Cedex France

**Keywords:** behavior, competition, conservation, cormorant, population trends, range shift, spatiotemporal analysis

## Abstract

Few long‐term, large‐scale studies have been conducted about the factors likely to explain changes in species abundance and distribution in winter. Range shifts are generally attributed to the climate change or land use. This study shows that other factors such as species protection and the ensuing increasing numbers of individuals and competition could be involved. It details the progressive conquest of France, the most important European wintering area for great cormorant, in three decades as its legal protection by the EU Birds Directive. It is based on 13 exhaustive national counts. Cormorants first occupied the farthest areas (Atlantic and Mediterranean lagoons, then larger rivers) from the main‐core European breeding area, with only progressive occupancy of the northeastern part later. This strategy mainly resulted from competition for optimal available feeding areas. Suboptimal areas (smaller wetlands harboring smaller night roosts, colder northeastern French areas) and progressive fragmentation of large night roosts into smaller, better located ones minimized flight costs. The coldest areas were occupied last, once other areas were saturated. Their occupancy was favored locally by the global climate change, but it played a minor role in these strategies. Both factors induced only a small NNE shift of the weighted centroid range of the wintering population (2.6 km/year) which mainly resulted from competition (buffer effect). Only the 2009 cold wave decreased the total number of wintering cormorants at the national scale, once the population had probably reached the carrying capacity of the country, while the previous cold waves had a minor effect. Comparatively, there was a greater SSE range shift of the weighted centroid of the breeding population (4.66 km/year). Range shifts of other recently protected species have been attributed to the sole climate change in the literature, but competition due to the saturation of usual wintering or breeding areas should be considered too.

## 1 INTRODUCTION

Numerous studies have shown changes in the abundance and distribution of bird species mainly during the breeding season, owing to habitat destruction (Dolman & Sutherland, 1995), protection of species and habitats (Deinet et al., 2013), and more recently the climate change or new feeding resources (Adriaensen, Ulenaers, & Dhondt, 1993; Feare, 1994; Fiedler, 2003; Lindström, Green, Paulson, Smith, & Devictor, 2013; Loonen & De Vries, 1995; Merkel & Merkel, 1983; Møller, Fiedler, & Berthold, 2010; Nilsson & Persson, 2000; Schmidt, 1998; Visser, Perdeck, Van Balen, & Both, 2009; Wu & Zhang, 2015; Zuckerberg et al., 2011). However, few long‐term, large‐scale studies have been conducted to minimize misinterpretations about different factors, particularly during the wintering season (Dalby, Fox, Petersen, Delany, & Svenning, 2013; Hughes, 2000; Lehikoinen et al., 2013; Valiela & Bowen, 2003; Walther et al., 2002). Several studies about range shift were based on large multi‐species databases of annual winter counts at large geographical scales. They ascribed the latitudinal range shift to the climate change (Maclean et al., 2008; National Audubon Society 2014; Niven & Butcher, 2009; Potvin, Välimäki, & Lehikoinen, 2016, Visser et al., 2009); But they did not take into account each individual life history and the entire annual cycle, which are important to understand the mechanisms that underlie shifts as a real response to the climate change (Elmberg, Hessel, Fox, & Dalby, 2014; Knudsen et al., 2011; Lehikoinen, Saurola, Byholm, Lindén, & Valkama, 2010; Paprocki, Heath, & Novak, 2014; Potvin et al., 2016). The latitudinal shift appeared to be mostly group or even species specific, and to depend on countries (it was higher under warming climates), with sometimes opposite directions according to species or seasons (Chen, Hill, Ohlemüller, Roy, & Thomas, 2011; La Sorte & Thompson, 2007; Niven & Butcher, 2009; Potvin et al., 2016). Although the buffer effect due to competition could affect dispersal strategies by driving birds to suboptimal areas due to higher habitat saturation (Krebs & Davis, 1978, Rushing, Dudash, Studds, & Marra, 2015; Pöysä et al., 2016), it was not taken into account in the debate about the global climate change. Therefore, a complementary study at the species level integrating the individual long‐term ecological record of the species at a large geographical scale is required. It is more particularly needed in the case of species showing a strong new population dynamics or recovering previous distribution areas as a result of species and/or habitat protection. This is even more relevant since the implementation of the U.S. Migratory Bird Treaty Act Protected Species (1972) and Bird Directive of the European Union (1979).

The continental subspecies of the great cormorant *Phalacrocorax carbo sinensis* has underwent one of the most greatest bird population booms and dispersals (along with the double‐crested cormorant *Ph. auritus* in the U.S., Wires & Cuthbert, 2006) in less than three decades and is therefore an interesting model for testing these changes (Marion & Le Gentil, 2006). In Europe, the marine subspecies *Ph. c. carbo* did not globally change its breeding distribution on the marine coasts of Iceland, Norway, the British Isles and northwestern France (with a newly described marine subspecies from Norway to France, *Ph. C. norvegicus,* Marion & Le Gentil, 2006). In contrast, protection and the increase in food resources due to eutrophication of waters caused the continental *P. c. sinensis* population to sharply increase. Once located in the refuge of the core area of the Baltic sea and the Netherlands, it extended to a large part of Europe (from around 5,000 breeding pairs in 1970 in northwest Europe to 191,000 in 2012, Van Eerden & Munsterman, 1995; Marion, 1997a, 2014b, Bregnballe et al. 2013), and its wintering population increased concurrently from probably less than 15,000 individuals in 1970 to 600,000 in 2013 in northwest Europe and north Africa. These wintering birds generated new breeding populations within the wintering area (De Juana & Garcia, 2015; Marion, 1997a), probably in relation to progressive saturation of the northern native breeding areas (Marion & Le Gentil, 2006).

**Figure 1:**
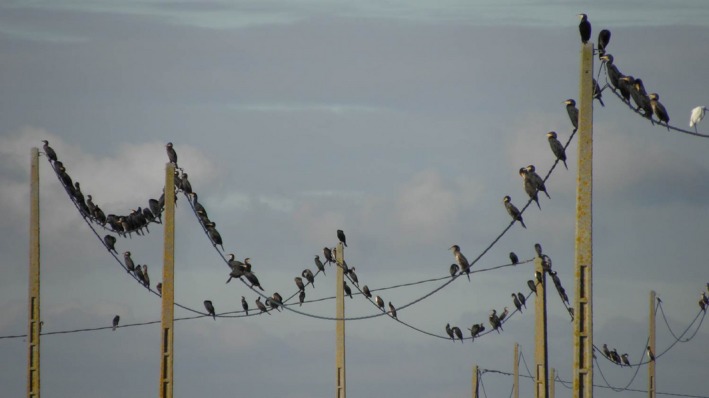
Night roost of great cormorants (P. Marion)

France is the main country for wintering and migrating cormorants in Europe. They mainly come from northern Europe (Frederiksen, Korner‐Nievergelt, Marion, & Bregnballe, 2018; Marion, 1995), and France is the only European country where their populations have been monitored by exhaustive and regular national winter counts using the same method (this is essential to describe changes in bird populations according to Elmberg et al., 2014). As such, France is particularly adapted for a long‐term study (33 years) of the rapid changes in cormorant distribution and numbers. In the daytime, cormorants are dispersed in groups or individually in the feeding areas (web of rivers, lakes, lagoons, sea‐coasts). By contrast, they are very social at night: they join a few permanent night roosts (Figure 1) which are generally close to or surrounded by water in sites preferentially undisturbed by humans (safety behavior, Marion, 1995). Similarly to colonies of herons, the radius of the feeding area and thus the mean distance of feeding flights (and associated cost of flight) are thought to be related to the number of birds in the roost, which varies from few birds to more than 2,000 (Marion, 1984, 1997a,b). The aims of this study were (a) to summarize the strategy of the conquest of France by wintering cormorants at different geographical scales, and (b) to test if changes were temperature dependent in the context of the global climate change or mere consequences of the global protection of the species in Europe since the 1970s, because protection induced a strong increase of the population and thereby probably increased competition for optimal areas. We tested (a) if the progressive occupancy of the country was geographically oriented; (b) if the optimal areas were occupied first until saturation and before suboptimal areas; (c) which respective roles temperature and water surface areas played in the characterization of optimal wintering areas for the species; (d) whether there was a change in the strategy of cormorant distribution (roost size) to minimize costs of flight and competition.

## 2 METHODS

Night roosts are generally occupied every year (which facilitates monitoring) except when strong disturbances occur, and most of them gather tens to hundreds of noisy cormorants. Night roosts with less than 10 birds are very rare. To discover the few new night roosts during each national census, counters followed the direction of straight flights of cormorants that flew directly to their night roost, and/or inspected the banks of rivers, lakes or sea coasts. Over 13 winters (named from each January) from 1983 to 2015, national censuses of all the night roosts were coordinated (by Loïc Marion, excepted in 1983 by Eric Pasquet) in the evening or more rarely early in the morning, with a network of counters who knew the local distribution of roosts very well. The roosts were counted simultaneously in mid‐January, when migration movements are usually minor. Such counts of night roosts were chosen to avoid underestimation (up to 50%, Marion, 1997c) of daytime counts in feeding areas through the International Waterbird Census and/or in day roosts. However, daytime counts were used in a few rare cases when night roosts were not counted because there were not enough counters or weather was foggy. When neither night nor daytime data were available in a given year, night roosts sizes were estimated by comparing numbers with those of neighboring roosts or neighboring “departments” (administrative French districts) between two successive national censuses. Such estimates only represented 0.5% to 2.87% of the national number of wintering cormorants (Marion, 2012a, 2014a, 2015a). Data were detailed in national reports for the French Ministry of the Environment (Pasquet, 1983; Marion, 1991, 1997c, 1999, 2001, 2003, 2005, 2007, 2009, 2011, 2012a, 2014a, 2014b, 2015a).

**Figure 2:**
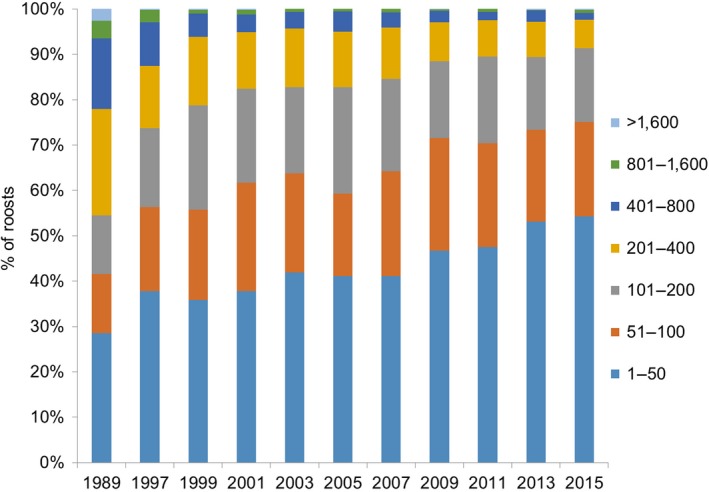
Changes in the proportions of the size classes of cormorant night roosts in France between 1989 and 2015

Population sizes during the exhaustive mid‐January counts were compared at different geographical scales: (a) the national level, (b) four main geographical areas: the coastal departments bordering the Channel, the Atlantic coast, the Mediterranean coast (including Corsica), and all other noncoastal departments gathered within an “inland area,” (c) ecological areas gathering the departments into 15 main watersheds, and (d) the 95 administrative departments. It was not a mere sampling of roosts, the whole population was counted, so that standard deviation across years is not needed. Population dynamics (the number of cormorants from all 13 censuses) is shown at the scale of the 15 watersheds, while the timing of the strategy of occupancy of the country is presented on national maps for each department but only based on a sample of annual counts (all the first counts from 1983 to 1997, and then every 6 years: 2003, 2009, and 2015). The detailed distribution of all night roosts according to their size is only presented for five national censuses (every 5–6 years from 1992 to 2015) as an example of the dynamics of individual roosts. While the exact number of cormorants was used in all analyses, we grouped for the Figure 2 the roosts into seven exponential classes according to their size (number of cormorants) from the smallest (<50 cormorants) to the biggest roosts (>1,600). The range shift of the wintering population over the study period was calculated for each census by multiplying the number of cormorants wintering in all occupied departments in mid‐January by the geographical coordinates (in degrees and decimal degrees) of their centre, and then dividing their sum by the total number of wintering cormorants in the country (“weighted geographical centroid” from Maclean et al., 2008). As a comparison, the range shift of the French breeding population of cormorants was calculated similarly from the localization of each colony over nine national counts coordinated by Loïc Marion, from 1983 to 2015.

When normality was not present, we used nonparametric tests. The role of temperature on the mean size of the wintering population of cormorants in January was calculated by comparing the average number of wintering cormorants in each department over the 13 censuses with the average temperature in January in each department (Spearman test, climate data from Météo France). The role of temperature in the chronology of the geographical conquest of France by wintering cormorants was tested by classifying the date of appearance of the first night roost in each department into six classes (up to 1983, 1989, 1992, 1997, 1999, and 2001), using ANOVA for independent series and then grouping 1989 and 1992 together. The role of competition for optimal feeding areas in the chronology of occupancy of the departments was tested using their water surface using Carthage database, version 2013. Optimal cormorant feeding areas are large open waters such as coastal lagoons, lakes, large rivers, and estuaries, while the secondary web of rivers only represents suboptimal feeding areas, as regards the fish stock and safety from humans generally present on the banks (Marion, 1983, 1995). According to the theory of competition among animals (Krebs & Davis, 1978), suboptimal areas are progressively used only after saturation of optimal feeding areas (Marion, 1997b). So we compared the correlations between the numbers of cormorants in each department and the water surface area, small rivers <50 m wide (suboptimal areas) excluded, over the successive national censuses. A decreasing correlation with time meant an increasing buffer effect of dominated birds rejected toward the suboptimal areas, owing to increasing competition and saturation in optimal areas. The precise distribution of cormorants in the feeding areas in the daytime was not available, so we did not directly correlate the number of cormorants with the types of feeding areas.

We investigated the global relations between cormorant numbers according to years, departments, water surface areas, and annual temperature in January using Generalized Mixed Effect Models (GLMs, R package “lme4”). More precisely, GLMs assuming Poisson's distribution were used to test if cormorant numbers were related to water surface area (fixed effect), temperature (fixed effect), year (fixed effect), and department (random effect). We then calculated the conditional and marginal pseudo‐*R* ^2^ coefficients of determination of GLMs to represent the variance explained by both fixed and random factors (R package “MuMin”). Analyses of variance (ANOVA) of the GLMs were performed using type 3‐ANOVA (R package “car”), and associated *p*‐values were calculated to test the significance of each explanatory variable. These statistical analyses were performed in the free and open‐source R platform (R version 3.4.2, R Core Team).

## 3 RESULTS

### 3.1 Population trends

**Figure 3:**
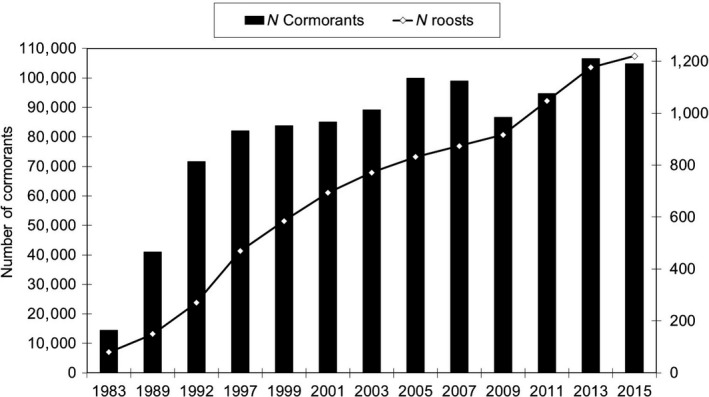
Mid‐winter (15 January) numbers of wintering cormorants in France between 1983 and 2015 (left axis), and numbers of night roosts at the same dates (right axis)

The numbers of wintering cormorants in mid‐January strongly increased between 1983 and 1992, and then progressively leveled off afterward, with a temporary decrease during the 2009 cold wave (Figure 3). The number of night roosts in mid‐January was globally correlated to the number of cormorants (Pearson's *r* = 0.929, *p* < 0.0013, *R* ^2^ 86.2%), but did not decrease in 2009.

### 3.2 Was the progressive occupancy of the country geographically oriented?

#### 3.2.1 Changes in distribution at different scales

**Figure 4:**
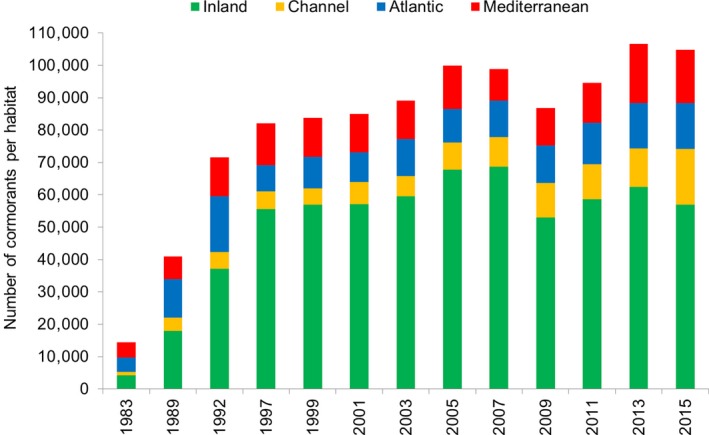
Distribution of wintering cormorants in France between 1983 and 2015 according to the four geographical areas

**Figure 5:**
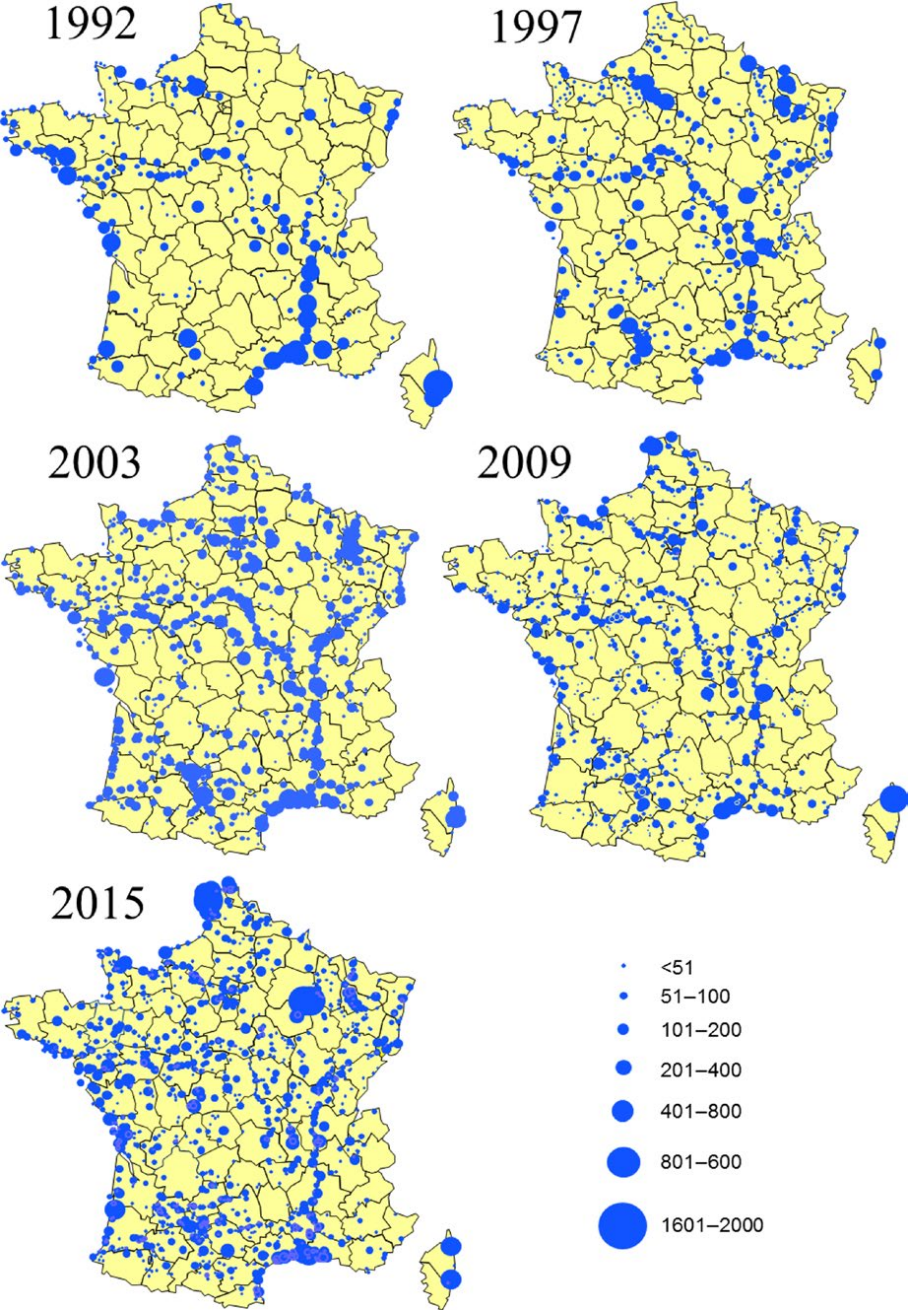
Sample maps (every 5–6 years) of all night roosts of cormorants in France according to their size in January between 1992 and 2015

In France, wintering cormorants mainly originated from northern European countries (continental subspecies from The Netherlands and Baltic countries, and marine subspecies from the UK, Marion, 1995). Strangely enough, they conquered France preferentially via the more distant wintering areas, that is, the Mediterranean and Atlantic coasts (Figure 4), despite the cost of flight. The English Channel was used by less than 10% of the birds until 2007. However, the wintering population on the Atlantic coast plummeted in 1997 (−51%), followed by a slow partial increase until 2003 and a leveling off around 11,000 birds until 2009, and a low increase again between 2011 and 2015 up to 14,200 birds. The Mediterranean coast (essentially lagoons) was also rapidly saturated in 1999 with around 12,000 birds, yet it sustained surprising increases in 2013 (18,265 birds) and 2015 (14,471 birds). These saturated pioneer areas were offset by a very strong increase in inland areas, which grew from 28% of the national population in 1983 to 68% in 1999, starting by the Rhône and Loire rivers that harbored one night roost every 40 km (Figure 5).

Then, the wintering population extended into inland area to other large rivers (the Seine, the Garonne) and progressively spread to the secondary webs of rivers with smaller roosts. Nevertheless, the proportion of cormorants wintering in inland areas leveled off until the 2009 crash (−21%, 53,333 birds); the crash was followed by a partial increase of the number of birds in 2011 and 2013, but with a new decrease in 2015 down to around 57,000 birds, a similar number as in 2001. Proportionally speaking, the importance of the inland area still decreased in 2013 and 2015, dropping to only 57% and 54% of the national numbers, respectively. The decrease was due to the increasing numbers on the Mediterranean coast and on the English Channel coast, mainly in the Pas‐de‐Calais and Nord departments that were unoccupied at the beginning of the survey, but occupied by 9,400 birds in 2015 (9% of the national population).

#### 3.2.2 Change of the weighted geographical centroid of the population

**Figure 6:**
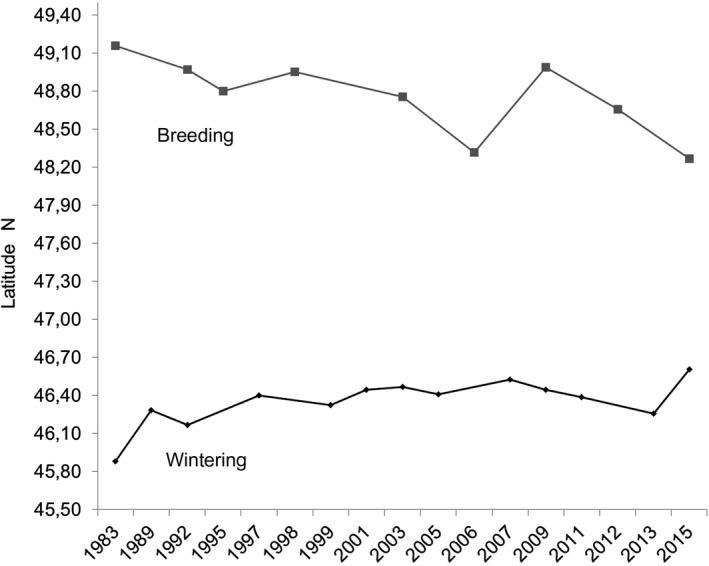
Change in the latitude of the weighted geographical centroid of wintering (a) and breeding (b) cormorants in France between 1983 and 2015

The weighted centroid of the wintering population in January shifted by 83 km (=2.60 km/year) in a NNE direction between 1983 (45°881N/2°213E) and 2015 (46°609N/2°466E), with a significant linear change between latitude and years (latitude N = 0.01414*year + 18.06, *p *< 0.0036, *R* ^2^ = 55.3%), despite an abnormal decrease of the latitude in 2013 (Figure 6). There was a strong correlation between the latitudinal weighted centroid and the number of wintering cormorants in January, 2013 excluded (Spearman *r* = 0.811 *p* < 0.007). However, when 2013 was included, it was not valid any more (Spearman *r* = 0.544, *p* < 0.06).

Comparatively, the weighted centroid of the breeding population shifted by 149 km (=4.66 km/year) in a SSE direction between 1983 (49° 159′N/−1.220W) and 2015 (48°270N/0°306E), which represented a shift of 104 km latitude eastwards and of 109 km longitude southwards. It was more regular for longitude over time (Figure 6: longitude E = 0.05812*year − 116,6, *p* < 0.000012, *R* ^2^ = 94,5%, latitude N = −0.02131*year + 91.41, *p *< 0.027, *R* ^2^ = 52.5%).

### 3.3 Were optimal areas occupied first until saturation and before suboptimal areas?

**Figure 7:**
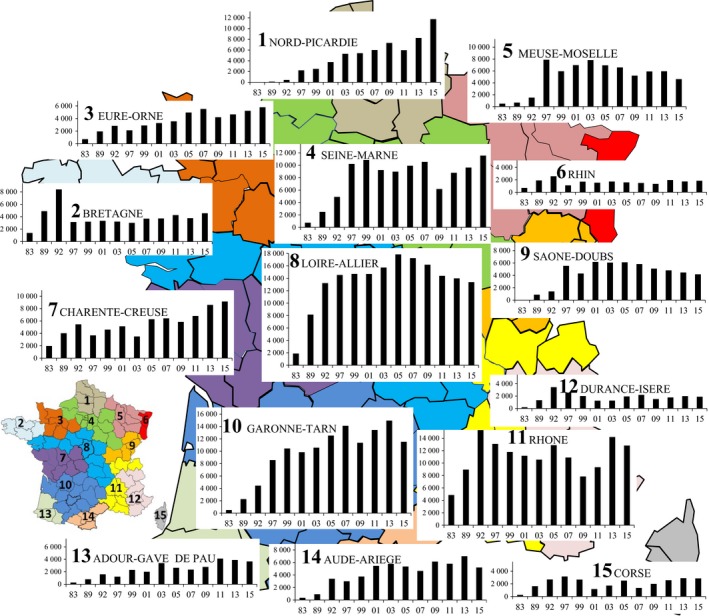
Historical record of the conquest of the 15 natural French fluvial areas by wintering cormorants between 1983 and 2015

At a large scale (three coastal areas and the inland area), the inland area was mainly occupied after the saturation of the south and west sea‐coasts (optimal areas) and then the northern coast (Figure 4). At a more detailed scale, the strategy of occupancy of the 15 main watersheds between 1983 and 2015 was similar (Figure 7). There was a rapid conquest of the areas representing the main eastern migration route mainly used by Baltic birds (n°6‐11‐12), and Brittany (n°2) representing the western migration route used by birds from The NL and the UK. All these populations reached their peak of numbers as early as 1992 and then declined, sometimes sharply (n°2‐11‐12‐15). The 1992 peak was also recorded in areas n°3, 7, and 13, followed by a decrease in 1997 and a subsequent increase. By contrast, the rapid transfer of birds from the declining areas or the additional migrating birds in France mainly concerned areas n°4 until 1999, n°9 and 14 until 2001, n°5 until 2003, n°8 until 2005, n°3 until 2007, n°10 until 2013, and more slowly and more progressively n°1 until 2015. The 2009 cold wave decreased the numbers of cormorants in all areas except n°2 and 14, with a higher impact in areas located in eastern and central France, but also in Normandy. Independently of normal annual fluctuations, there was a sharp contrast between (a) areas where the numbers of wintering cormorants durably leveled off after the decrease that followed the 1990s peak in numbers (n°2‐4‐6‐11‐12), and (b) areas where the numbers of wintering cormorants regularly decreased (n°5‐8‐9), or conversely (c) those in which the numbers regularly increased (n°7‐10‐13‐14 and above all n°1 = Nord‐Picardie, which was first avoided but finally represented the third most occupied area in France).

**Figure 8:**
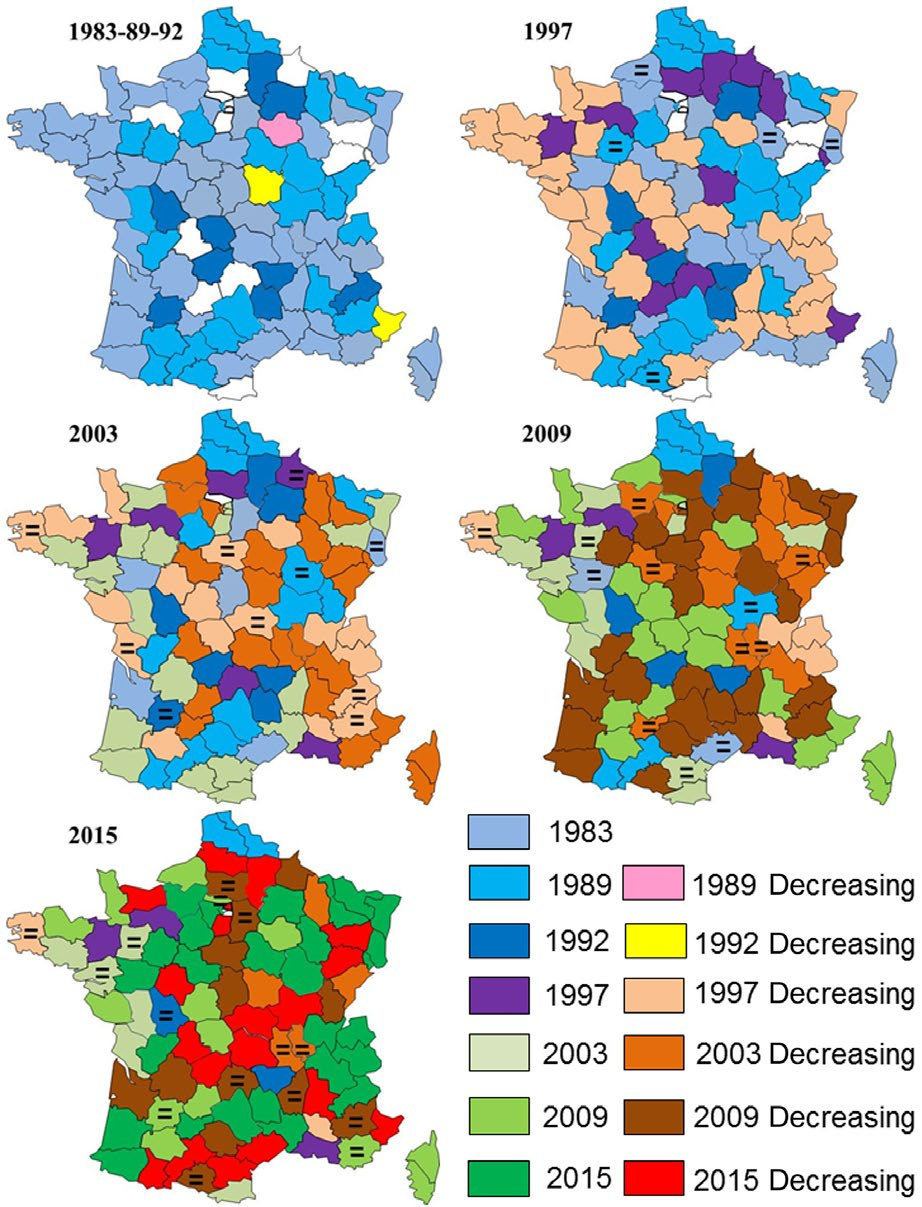
Historical record of the occupancy of each of the 95 French departments from 1983 to 2015. The color used for the first national census in 1983 shows the presence of wintering cormorants at this date; it remains the same (similarly to the other colors of the left column in the legend) for the following censuses as long as cormorant numbers kept increasing. The colors of the right column were used when the numbers decreased by >10% (and remained the same for the following censuses as long as cormorant numbers kept decreasing). The sign “=” was used when numbers leveled off (±10%). A following increase or decrease >10% after such a leveling off is shown by a color change in the corresponding year

Figure 8 analyzes the strategy of occupancy of France at the lowest geographical scale, that is, the 95 administrative departments. Forty‐two departments were already occupied in 1983, while 24 new ones were occupied in 1989 and 9 in 1992, notably inland departments in southeastern and northeastern France, yet with still 10 of them unoccupied in that latter area in 1992 out of the 15 unoccupied ones at the national level. We analyzed the respective role of temperature and wetland areas at this geographical scale.

### 3.4 What respective role did temperature and water surface areas play in the characterization of optimal wintering areas for this species?

#### 3.4.1 Role of temperature in the progressive occupation of wintering areas

The cold wave of the 1997 winter caused the first important change in the dynamics of numbers in France after 14 years of increasing numbers (Figure 7): the number of cormorants decreased in 30 departments and leveled off in 5, most of them in the northwest and southeast areas, even before the total conquest of the country (nine departments still remained unoccupied, essentially in the coldest northeast area).The situation was partly similar during the 2003 cold wave, but there was only one unoccupied department, and above all the population dynamics became geographically much more complex than in 1997, with opposite dynamics in most of the neighboring departments instead of regional tendencies as observed previously.

Populations dramatically decreased during the 2009 cold wave, when the distribution of declining or stabilized departments practically concerned the whole country except the northwestern part, with again increasing heterogeneity in dynamics among departments (increase versus decrease >10% and vice versa). This heterogeneity was noted only in 3.12% of the departments between two successive censuses until 1992, in 43.74% of them between 1997 and 2007, and in 49.47% of them between 2009 and 2015. Only one department (Nord) kept the same dynamics since its first occupancy, with a continuous increase in the number of cormorants.

**Figure 9:**
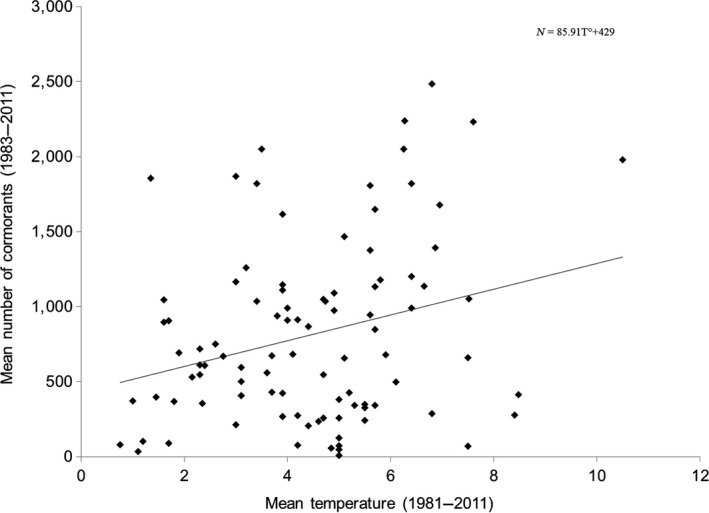
Relation between mean temperatures in January between 1981 and 2010 in France and the mean numbers of wintering cormorants in each department between 1983 and 2011 (*N* = 95)

The slope of the mean temperature in January during the studied years was not significantly different from 0 (*p* < 0.84). The mean number of wintering cormorants between 1983 and 2015 in each department was little correlated to this mean T° (Figure 9, Spearman's *r* = 0.257, *p* < 0.013), with a low *R* ^2^ (6.6%).

Nevertheless, the departments occupied as early as 1983 were significantly warmer (mean 1983–2015 January temperature 4.87°C) than those occupied later between 1997 and 1999 (3.51°C, ANOVA: *F* = 3.528, *n* = 88, *p* < 0.034, post hoc Tukey's test *p *< 0.039). The departments conquered during the mid‐period (1989–1992) had an intermediate temperature (4.14°C) which did not significantly differ from the temperature recorded at the other dates of occupancy. The occupancy of the seven departments occupied from 2001 was not related to temperature.

#### 3.4.2 Role of competition for optimal feeding areas

**Figure 10:**
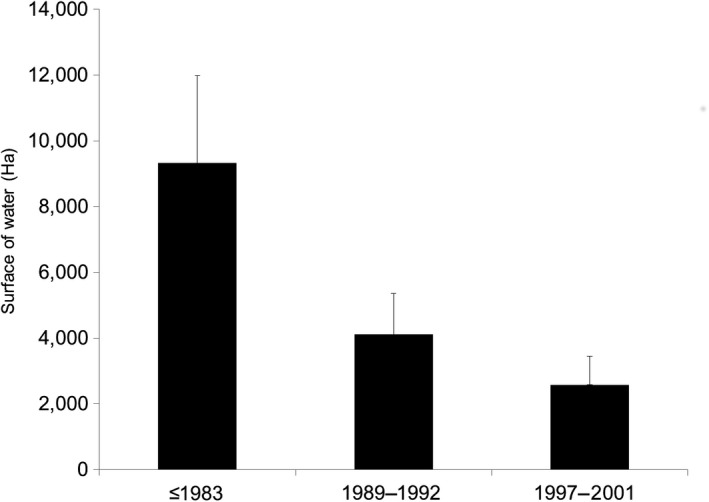
Chronology of occupancy of the French wintering areas of cormorants according to their average surface of water

The mean number of wintering cormorants in each department between 1983 and 2015 was largely related to the surface area of water bodies, small rivers <50 m wide excluded (Spearman's *r* = 0.545, *p* < 0.00001, *R* ^2^ = 29.7%). However, this relation decreased regularly from a maximum in 1992 (Spearman's *r* = 0.595, *p* < 0.00001, *R* ^2^ = 35.4%) to a minimum in 2013 (Spearman's *r* = 0.408, *p* < 0.00008, *R* ^2^ = 16.6%), showing indirectly the increasing use of the suboptimal web of small rivers. Wintering cormorants first occupied (≤1983) the optimal areas (the departments with the largest surfaces of water bodies: $\overset{¯}{X}$ = 9325 ha, *N* = 46) and then suboptimal areas up to 1992 ($\overset{¯}{X}$ = 4,113 ha, *N* = 27), and finally the least optimal areas up to 2001 ($\overset{¯}{X}$ = 2,582 ha, *N* = 22, Figure 10, Kruskal–Wallis index 29.94, *p* < 0.00001). The time‐course of occupancy of the 95 departments was much more related to their water surface areas used as a criterion of feeding resources (Spearman's *r* = −0.575, *p* < 0.00001, *R* ^2^ = 33.1%) than with climate.

### 3.5 Was there a change in the strategy of cormorant distribution (roost size) to minimize costs of flight and competition?

There was a change in the strategy of wetlands occupancy after 1989, when the mean annual rate of the increasing number of night roosts became higher than the rate of the number of individuals (Figure 3).

**Figure 11:**
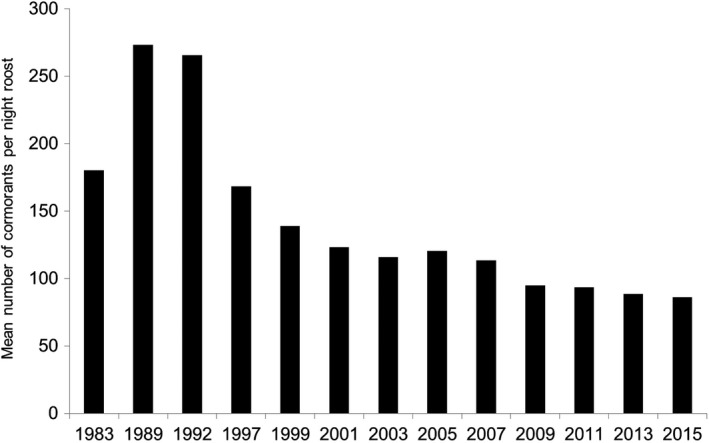
Mean size of night roosts of cormorants in France between 1983 and 2015

The mean size (=number of cormorants) of night roosts was negatively correlated to the total number of cormorants (Pearson's *r* = −0.896, *p *< 0.0019, *R* ^2^ 80.2%) and above all to the number of night roosts (Pearson's *r* = −0.978, *p* < 0.0007, *R* ^2^ 95.7%). However, it strongly increased between 1983 and 1989 and then strongly and regularly decreased afterwards (Figure 11).

Such a decrease corresponded to a regular decrease of the largest roosts: in 1989, 45.45% of the roosts harbored between 201 and >1,600 cormorants, vs. only 8.49% in 2015, while the biggest roosts had practically disappeared (Figure 2). These decreases benefited to small roosts (<51 individuals) which reached 53.47% of the roosts in 2015 vs. 28.57% in 1989, and to middle‐sized roosts (51–100 individuals) that reached 20.50% in 2015 vs. 13% in 1989.

### 3.6 Global analysis of the respective factors involved in cormorant distribution: geographical area, year, water surface area, and temperature

GLMs results showed that year, water surface area, and temperature explained 43.95% of the variance, and this percentage increased up to 71.93% when taking into account the department’ random effect (Model 1, Table 1). GLMs (Models 2 to 7) showed that (a) year, (b) department, (c) water surface area, and (d) temperature (in decreasing order) mainly explained the size of the populations. Models 5‐6‐7 showed that year, wetland surface area, and temperature explained 37.84%, 13.68%, and 0.44% of the variance, respectively. For each GLM model (from 1 to 7), all tested variables were associated to significant *p*‐value (<0.001) following type‐3 ANOVA tests.

**Table 1:** Variances explained (%) by fixed factors (FR) and random factors (RF) in the GLMs

Models	Variance explained by fixed factors (%)	Variance explained by fixed factors and random factors (%)
Model 1: Cormorants ~ Temperature (FR) + Wetland surface (FR) + Year (FR) + departments (RF)	43.95	71.93
Model 2: Cormorants ~ Wetland surface (FR) + Year (FR) + departments (RF)	43.87	71.89
Model 3: Cormorants ~ Temperature (FR) + Year (FR) + departments (RF)	37.87	68.89
Model 4: Cormorants ~ Temperature (FR) + Wetland surface (FR) + departments (RF)	14.11	56.99
Model 5: Cormorants ~ Year (FR) + departments (RF)	37.84	68.87
Model 6: Cormorants ~ Wetland surface (FR) + departments (RF)	13.68	56.78
Model 7: Cormorants ~ Temperature (FR) + departments (RF)	0.44	50.15

## 4 DISCUSSION

This study investigates the mechanisms of expansion of cormorant wintering populations in France and the changes in their distribution between 1983 and 2015 according to climate change and to competition for optimal areas. Our results show that year and department effects significantly explained the progressive evolution of cormorant site occupancy in France (68.87%). This evolution was also related to water surface areas (13.68%) and therefore to competition for optimal habitats. Although the role of temperature (0.44%) was significant, it should be mitigated to explain the shift of cormorant distribution across France. This being explained, we finally discuss the role of protecting areas, shooting, and behavior in roost atomization.

### 4.1 Mechanism of population expansion and role of competition for optimal habitats

In accordance with the Island biogeography theory of MacArthur and Wilson (1963) and subsequent studies about metapopulation models (Levins, 1969; Wiens, 1976), new areas are generally conquered via successive, more and more distant steps from the starting point. There is a hierarchy in the chronology of occupancy from optimal to suboptimal areas, with suboptimal areas being becoming occupied once optimal areas are saturated (Krebs & Davies 1978, Marion, 1984, 1989, 1997b; Rushing et al., 2015). The Island theory concerns breeding populations, with new individuals (propagules) produced in the successive conquered zones that in turn participate to progressive geographical expansion. The case of wintering cormorants is different because they usually return to their native area located in northern Europe each following spring. Among birds in general the wintering area is usually quite far from the breeding area for climatic reasons. However, we can admit that a similar pattern to the Island theory could occur with the progressive geographical conquest of potential (more distant) wintering areas by cormorants for obvious energy costs of flight because the breeding and wintering populations successively increased in Europe after protection of cormorants by the EC Bird Directive in 1979 (Marion, 1997b; Van Eerden & Munsterman, 1995), as numerous bird species did once legally protected.

Moreover, these wintering cormorants also generated new inland breeding populations, notably in France (up to 118 colonies totaling 7,248 breeding pairs in France in 2015, in addition to the oldest marine population that reached 2,124 breeding pairs distributed across 34 colonies, Marion, 2015b). But these French breeding populations contributed little (an estimated 14%) to the wintering population in France because part of the birds wintered abroad (Marion & Marion, 2018). These colonies essentially occupied the northern half of France, where the ecological niche of temperature for breeders (a relatively cold climate) favored this situation (Marion, 2014b).

The results of the present study do not follow the Island theory: new inland breeders first bred in Brittany, far from the core breeding area of the continental subspecies *Ph. c. sinensis* located in northern Europe, and the wintering population first preferentially occupied the most distant wintering areas such as the Atlantic and Mediterranean coasts, while occupancy of the northeastern areas proximate to the northwest European breeding population occurred well after (Figures 4, 5 and 7). This raises the issue of the suitability and carrying capacity of the different areas: Were they really much lower in the departments of Nord‐Picardy (n°1 in Figure 7) or in areas n°5 and 9 than on the Atlantic and Mediterranean coasts in the 1980s? Moreover, what can explain the strong increase of the numbers of wintering cormorants in Nord‐Picardy in the 2000s, in ecological habitats that existed in the 1980s when cormorants flew over them during migration, and in where there is no known reason for a recent increase in food resources? One explanation is that the southern and western optimal wintering areas, which had larger wetland surfaces, became saturated, and this induced an increasing shift to suboptimal northern areas where the availability of food resources was lower (smaller water surfaces) and more variable (cold wave, see below).

The cormorants displaced to northern areas were probably young birds (bird dominance hypothesis, Gauthreaux, 1982; see also Rushing et al., 2015 for American redstarts *Setophaga ruticilla*), supposed to be socially dominated by adults. The recent native adult French cormorants of the Lac de Grand‐Lieu did indeed migrate further south than young birds (Marion & Marion, 2018). Yet, Van Eerden and Munsterman (1995) and Bregnballe et al. (1997) observed the opposite about Dutch and Danish cormorants in the 1980s and 1990s: adult males remained close to the breeding grounds, whereas juvenile females migrated further south to the Mediterranean region.

Such an increase in wintering in northern areas was also recently observed in increasing populations of other birds with an extended wintering range such as the greylag goose (*Anser anser*) or the great crested grebe (*Podiceps cristatus*) in The Netherlands (Adriaensen et al., 1993; Loonen & De Vries, 1995; Nilsson, 2006), or the European spoonbill in France (Caupenne & Marion, 2015); for this latter species, it could also be due to saturation of southern wintering areas, and to the climate change that decreased the risk of wintering northernmost for many species (Fiedler, 2003; Issa & Muller, 2015). The survival rate of spoonbills became lower in the usual main southernmost wintering areas than in the northernmost wintering areas (Lok, Overdijk, Tinbergen, & Piersma, 2011).

In addition to the saturation of wintering areas, depletion of food resources may also have occurred in some southern areas as it did for other bird species (Zuckerberg et al., 2011). For example, the gulf of Gabès (Tunisia) used to be a hotspot for wintering cormorants in the 1980s (25–30,000 cormorants in 1980–1985, Van Eerden & Munsterman, 1986), three times more than in 2003 (Van Eerden et al., 2011) and 2013 (M‐A Dakhli, pers. com.).

### 4.2 Changes in migrating routes and wintering areas due to competition between geographical populations

The decrease in wintering cormorants along the Atlantic coast after 1992 (Figure 7), although in an optimal area for the species, participated to the NNE shift range of the national population and was probably due to competition between the two marine and inland subspecies. Danish cormorants initially migrated mainly through eastern routes in the 1980s and 1990s, but migrated further west afterwards (Frederiksen et al., 2018; Marion, 1995). Maybe there where under the pressure of competition with new northeastern populations migrating for the first time, which could also partly explain the decreasing numbers of cormorants in Tunisia. In western France, Danish cormorants competed with Dutch cormorants, which had previously evicted British cormorants *Phalacrocorax carbo carbo* from the French sandy Atlantic coasts (Marion, 1983, 1994, 1995). Unlike the *carbo* subspecies, *sinensis* birds did not use the marine habitat itself (Marion, 1983, 1995), so the available feeding area was reduced, and consequently the number of cormorants.

Such fluctuating competition between subspecies and probably geographical origins within a same subspecies (Danish and Dutch populations) has not been observed in other bird species. It was also recorded in the breeders of the pioneering and largest French colony of cormorants at the Lac de Grand‐Lieu (Loire Atlantique department). The colony was initially created by wintering birds belonging to the continental *Ph. c. sinensis* subspecies, but rapidly invaded by the marine subspecies *Ph. c. carbo* (Marion & Le Gentil, 2006). The situation became even more complex with the recent use of inland areas up to 300 km from the sea by the marine subspecies during wintering (Fonteneau & Marion, 2011; Fonteneau, Paillisson, & Marion, 2009). These changes between eastern and western migrating routes could also explain the recent and strong use of Spain as a wintering area, once France became progressively saturated and exported part of its own migrating birds toward Spain. Spain became the second most important wintering area in Europe after France in 2003 with about 75,000 cormorants (Del Moral & de Souza, 2004; Van Eerden et al., 2011) vs. around 12,000 in 1990 (Troya & Bernués, 1990). An increase in *sinensis* birds along the western migrating route was also observed down to Morocco where they competed with African populations of *Phalacrocoras carbo maroccanus* and *Ph. C. lucidus* (A. Qninba, pers. com.). Changes in migrating routes have rarely been observed in other birds (Gramet & Dubaille, 1983; Merkel & Merkel, 1983; Feare, 1994; Marion & Marion, 1994; Mewes, 1996; Schmidt, 1998; Fiedler, 2003; Todte et al., 2010; Ławicki, 2014).

### 4.3 Role of the climate change in the range shift

Globally, the mean January temperature over the three 1981–2010 decades contributed marginally to the mean number of wintering cormorants in each French department according to the GLMs models. However, this mean January temperature partly explained the time‐course of early (1983) and relatively late (1992–1999) department occupancy, but did not explain the occupancy of the last seven departments. Rather than the mean January temperature over the whole period, the climate risk (cold waves) in the north‐east area may have prompted cormorants to favor the Atlantic and southern areas from the start. The global climate change over the three decades of the study probably had a relatively small effect on the shift of the weighted geographical centroid in a northeasterly direction (Figure 6). The same was true for several waders in Europe (Maclean et al., 2008; Pavón‐Jordán et al., 2015) and 177 bird species in the USA; 79 bird species even shifted south (Niven & Butcher, 2009), like the breeding population of cormorants in France from 1983 to 2015. At the French national scale, the mean annual temperature became warmer than the mean temperature over the 1961–1990 period, but mainly locally. For instance, there were 75% of the years with more than 80 days with negative daily temperatures in Lorraine (eastern France) over the 1951–1970 period, only 45% over the 1971–1990 period, and 17% over the 1991–2013 period. Among the three moderate cold waves during that last period (93 days <0°C in 1996–1997, 87 in 2002–2003, and 80 in 2008–2009), only the latest one paradoxically had a strong effect on the numbers of wintering cormorants in France (−12.5% as compared to the previous census in 2007, Figure 3), even if the very high mean annual increasing rates observed until 1992 stopped in 1997. Again, the 2003 cold wave did not impact the number of cormorants, unlike in northern countries (Van Eerden et al., 2011). No relation was highlighted over the whole period between the annual number of negative daily temperatures in France and the number of wintering cormorants between 1983 and 2015 or with the latitude of the weighted geographical centroid.

The only strong effect of the 2009 cold wave in France, and the low recovery during the relatively cold following winters, could be explained by two complementary factors: (a) during the previous cold waves, the occupancy of northeastern France was not important enough to imply a visible impact on the population of wintering cormorants *via* mortality or migration abroad; (b) the saturation of the country around 100,000 cormorants from 2005, reflecting the hypothesized carrying capacity of the country, implied fewer possibilities of refuge in other areas, including Spain where the wintering population also seemed saturated around 70,000–79,000 cormorants, as compared to its under‐saturation before 2005. Paradoxically, there was a strong and unexpected increase of the number of wintering cormorants in January 2013, while the latitude of the weighted geographical centroid was abnormally low (Figure 6), with the greatest use of the Mediterranean area since 1992 (Figure 4, see also areas 10–11 in Figure 7), as if the previous cold waves had played a role in the choice of following wintering areas for survivors.

Numerous studies on the climate change hypothesized a climate niche and thus predicted that future decreases in numbers in southern areas would be offset by increases in northern areas for many species (Harrison et al., 2006; Pavón‐Jordán et al., 2015; Thomas et al., 2004), or in wintering areas (Barbet‐Massin, Walther, Thuiller, Rahbek, & Jiguet, 2009). However, according to Braunisch et al. (2013) such species distribution models could suffer from uncertainty because variables are often selected arbitrarily and their selection is difficult to control using contemporary information. But the numbers of cormorants did not decrease in southern areas, and neither did the numbers of wintering waders in Europe (Maclean et al., 2008). In their long‐term study about wintering waders in Europe, these authors attributed the increasing northeastern weighted geographical centroid to the climate change, and so did Niven and Butcher (2009) about a global study of wintering bird species in the USA, but this was not the case for European dabbling duck species (Dalby et al., 2013). The present study on cormorants shows that the role of the climate was minor as compared to the protection of the species. Protection allowed for a strong increase in the breeding and consecutive wintering populations, with a time course of occupancy of the country largely more related to water surface areas (13.68% of the variance explained) than to climate (0.44%, Table 1). As a result, competition among individuals increased, forcing the dominated birds to use the suboptimal northern wintering areas and the secondary webs of watersheds. They did so despite the higher risk of mortality from unpredictable cold waves, likely to deplete the population only when the carrying capacity of the habitats reached the saturation level.

### 4.4 Role of protected areas and shooting

Additionally, the increasing numbers of protected areas (hunting‐free reserves) in France over the study period did not explain the increase in the wintering population of cormorant, although it did so for some species (Madsen & Fox, 1997). Protected areas played a minor role in the distribution of winter roosts, and their role was more marked in breeding colonies (Marion, 2015b). A complementary analysis will be needed about fish resources to assess the carrying capacity of the country (Marion, in prep.), to try and explain why the level of 100,000 cormorants has been (momentarily?) overrun since 2013. The shooting of cormorants in France, from 5.55% of the wintering population (in January) in 1996 to 43.51% in 2013, had no effect on the dynamics of the wintering populations from winter to winter at the department scale (Marion, 2012b; Marion, in prep.). The same pattern occurred in Bavaria (Keller & Lanz, 2003), England (Chamberlain, Austin, Newson, Johnston, & Burton, 2013), and Denmark (Bregnballe, Hyldgaard, Clausen, & Carss, 2014): shot cormorants were rapidly replaced, probably mainly by young birds because adults tend to go back to the same wintering sites (Reymond & Zuchuat, 1995; Yésou, 1995).

### 4.5 Role of behavior in roost atomization

The behavior of cormorants (social attraction according to their geographical origin, fidelity to their wintering site, competition between sexes and ages, decreasing fear of humans…cf. Marion, 1994, 1995; Reymond & Zuchuat, 1995; Van Eerden & Munsterman, 1995) also played an important role in their strategies of habitat use. First (until 1989), the mean size of night roosts strongly increased, then the situation reversed (Figure 11), while the increasing rate of the number of roosts became higher than the rate of the number of cormorants (until 2011). This “atomization strategy” (decrease of the biggest roosts, Figure 2) followed the same pattern as the numbers of breeding grey heron colonies in France after the species was first protected in 1975 (Marion, 1997b; Marion & Marion, 1987; Marion, Van Vessem, & Ulenaers, 2000). When wintering cormorants (or breeding grey heron) occupied France again once they were legally protected, safety still outweighed for a time all the other factors for the priority choice of big pre‐existing roosts. These roosts played the role of a safety index as compared to creating new but potentially unsafe roosts in vacant areas. When the birds became progressively less afraid of humans and used less safety sites, the biggest roosts were gradually fragmented into many smaller and smaller roosts. Smaller roosts were better adapted to the distribution of feeding resources, with optimized costs of feeding trips. This strategy allowed for a strong increase in the wintering or breeding populations (Marion, 1997a,b; Marion & Marion, 1987). Paradoxically, the protection of these species caused their biggest roosts or colonies initially used as refuges to decrease.

## 5 CONCLUSION

The case of wintering cormorants shows that within a few decades protection can logically induce an increase in the population that generates increasing competition for optimal feeding areas. The impact of the climate change could differently affect the dynamics and distribution of populations according to the saturation of optimal habitats. If there is a geographical northsouth gradient of optimal–suboptimal habitats, competition among individuals may induce a similar or even a higher northern range shift than the climate change does, or induce an opposite shift with differences between breeding and wintering populations. Both factors (saturation/climate) should be studied simultaneously from long‐term, large‐scale databases to avoid misinterpretations about the impact of the climate change. Competition between geographical origins, including subspecies, could also induce changes in migration routes and cause competition to shift elsewhere. The heterogeneity of local situations (increasing vs. decreasing trends in the numbers of individuals) strongly increased with the global increase in the population and with the degree of saturation of habitats over time. The effect of the climate (cold waves) on population size could be higher in saturated populations, but contributed marginally to the distribution of cormorant over the three 1980–2010 decades. The example of the great cormorant could be extended to many other bird species protected in the 1970s or 1980s by the U.S. Migratory Bird Treaty Act Protected Species or the European Union Bird Directive. For these bird species, range shifts have mostly been only attributed to the climate change in the literature without taking into account competition due to the saturation of usual wintering or breeding areas.

## AUTHORS CONTRIBUTION

LM conceived and designed the study, collected data, performed analyses except GLMs and wrote the manuscript and made figures; BB performed GLMs analyses.

## CONFLICT OF INTEREST

None declared.

## DATA ACCESSIBILITY

Species data: available through Dryad (http://datadryad.org/).
